# Giant Cell Tumor of the Anterior Arc of the Rib: A Case Report

**DOI:** 10.7759/cureus.58679

**Published:** 2024-04-21

**Authors:** Manu Srinivas, Prabhat Nichkaode, Bijay Sharma, Shriya Haval

**Affiliations:** 1 General Surgery, Dr. D. Y. Patil Medical College, Hospital & Research Centre, Dr. D. Y. Patil Vidyapeeth (Deemed to be University), Pune, IND; 2 Surgery, Dr. D. Y. Patil Medical College, Hospital & Research Centre, Dr. D. Y. Patil Vidyapeeth (Deemed to be University), Pune, IND

**Keywords:** giant cell tumor, hand swelling, bone benign tumor, bone lesion, metacarpal lesion, aneurysmal bone cyst, rare site, anterior arch of rib, anterior chest wall

## Abstract

Tumors that develop on the chest wall are usually rare. This case report highlights a rare occurrence of a giant cell tumor originating from the anterior arch of the fourth rib. The patient, a 21-year-old male, presented with a bulging mass that had been gradually increasing in size over an eight-month period, reaching dimensions of 12 x 8 cm. Despite the noticeable swelling, the patient reported no associated pain or discomfort and denied any history of weight loss or trauma. The absence of chest pain or cardiovascular symptoms distinguished this case from other chest wall pathologies. This report underscores the importance of considering rare entities such as giant cell tumors in the differential diagnosis of chest wall masses, especially in cases where clinical presentation and patient history do not align with more common conditions.

## Introduction

Giant cell tumor (GCT) of the anterior arc of the rib is a rare condition, accounting for a small percentage of all GCTs [1]. These tumors typically arise from the bone tissue of the rib, specifically from the anterior arc, and are characterized by the presence of multinucleated giant cells within a stromal cell-rich background [2]. While GCTs can occur in various bones throughout the body, their occurrence in the anterior arc of the rib presents unique diagnostic and management challenges.

GCT is a benign tumor that typically manifests in middle-aged or older individuals, with a slight predilection for females [3]. While the knee joint is the most common site for these tumors, accounting for over half of all cases, tumors affecting the chest wall are rare [4]. Specifically, GCTs occurring in the rib are even less common, with reported frequencies of less than 1% [5].

Given the unusual location and low incidence of GCT in the rib, comprehensive diagnostic evaluation, including imaging studies such as X-rays, CT scans, and MRI, is necessary to characterize the lesion and ensure effective treatment [6]. Additionally, biopsy and histopathological examination are crucial for confirming the diagnosis and distinguishing GCT from other chest wall lesions or malignancies.

## Case presentation

**Figure 1:**
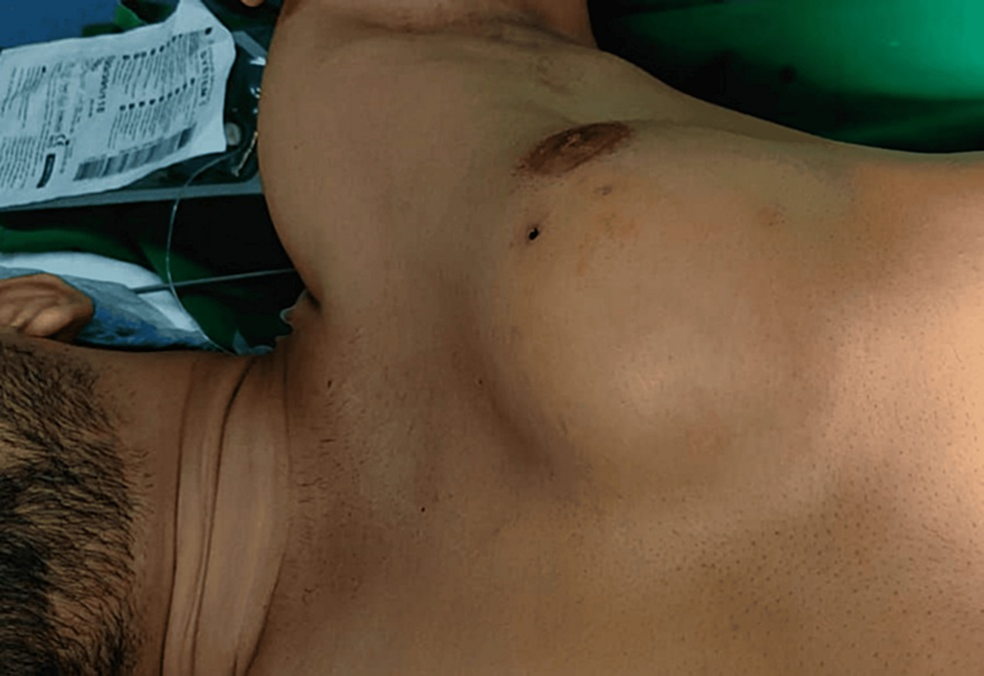
Clinical picture showing the left chest wall

A 21-year-old male patient presented with a swelling over the left chest that had been present for eight months, initially measuring 5 x 6 cm but subsequently enlarging to 12 x 8 cm (Figure 1). The patient reported no complaints of chest pain. Additionally, there was no history of weight loss or trauma. The patient's pulse rate was 90 per minute and blood pressure was 120/80 mmHg. Physical examination revealed an oval-shaped swelling measuring 12 x 8 cm, spanning the left anterior chest wall and involving ribs 4, 5, and 6. The skin surrounding the swelling appeared normal and could be pinched, while the mass itself was firm and fixed to the chest wall. The nipple-areola complex showed no abnormalities and no palpable lymph nodes were detected in the left axilla. Standard blood tests yielded normal results.

**Figure 2:**
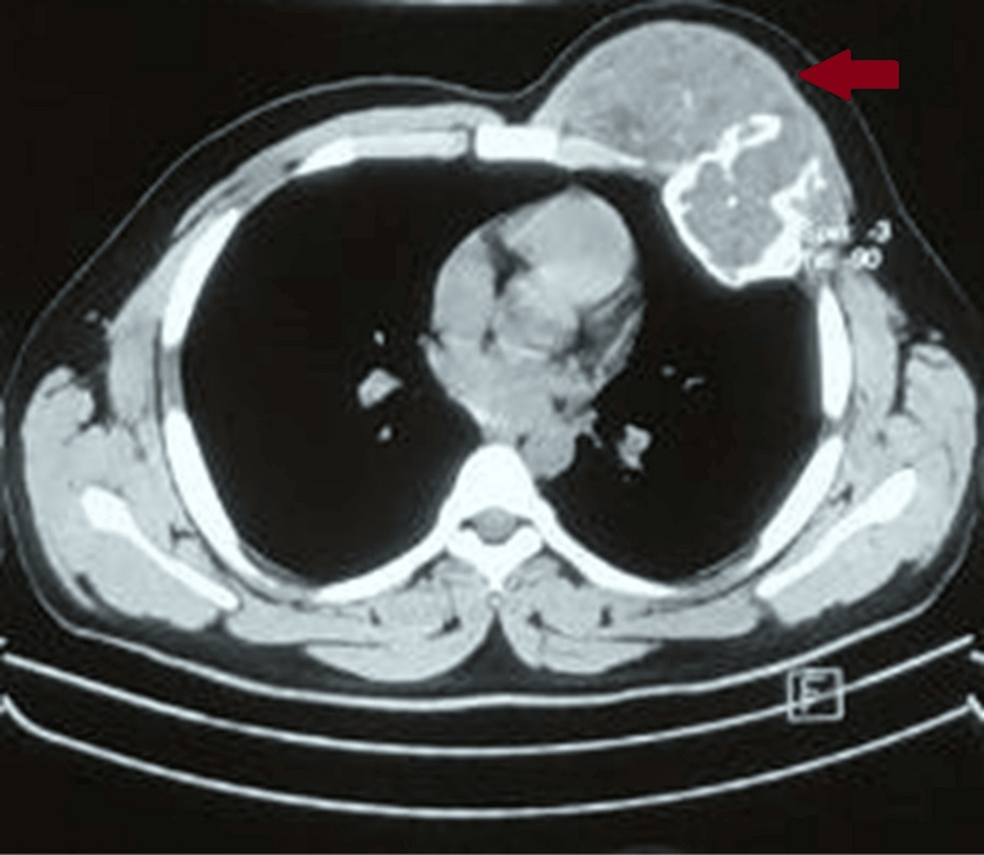
CT scan of the chest CT scan showing growth from the left chest wall (indicated by red arrow).

The chest computed tomography scan revealed a tumor affecting the muscles in the anterior chest region, accompanied by a large, well-defined, multilobulated swelling involving the anterior aspect of the fourth rib, with a slightly increasing soft tissue component measuring 6 x 5 x 6 cm (Figure 2). Both lung parenchyma appeared normal, and there were no calcifications within the lesions. The MRI of the chest showed involvement of the subcutaneous plane, myofascial plane, thoracic cage, and intra-thoracic area, characterized by well-marginated, lobulated lesions with heterogeneous signal intensity along the anterior left thoracic wall. Fine needle aspiration cytology (FNAC) did not provide a definitive diagnosis. However, biopsy results confirmed the presence of a GCT. Following resection, the serum acid phosphatase level decreased to 6.5 IU/L, significantly lower than the typical range of 10.2 IU/L.

**Figure 3:**
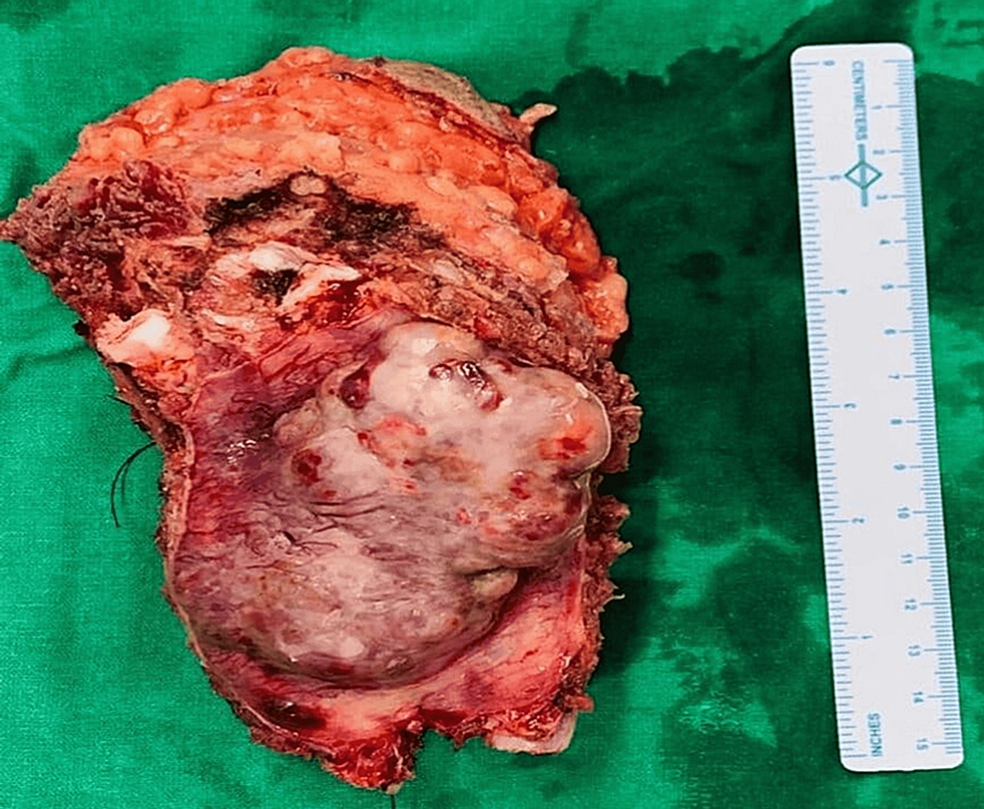
Postoperative excised region

**Figure 4:**
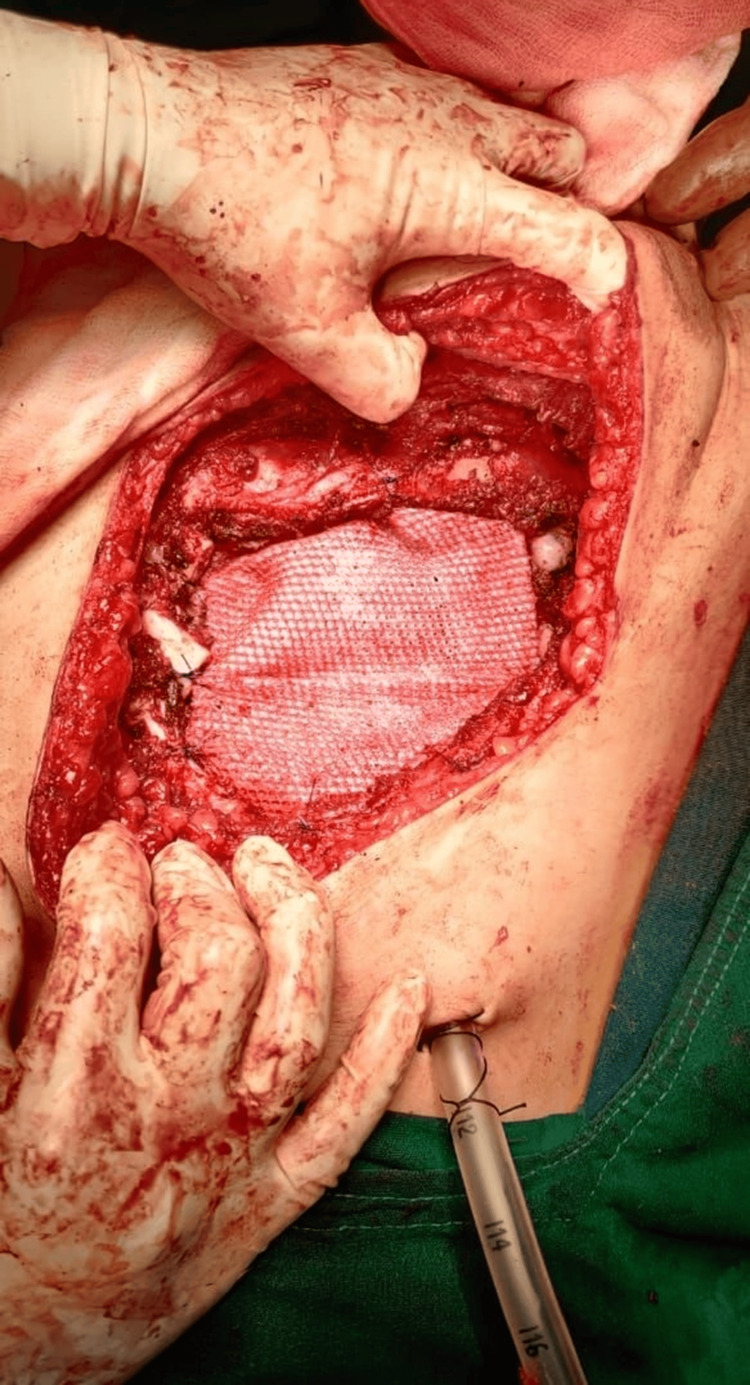
Polytetrafluoroethylene (PTFE) mesh placement

The patient underwent a significant local excision procedure, which involved removing the affected rib along with one rib above and one rib below it (Figure 3), followed by the placement of polytetrafluoroethylene (PTFE) mesh to reinforce the chest wall (Figure 4). Subsequent to tumor removal, reconstruction of the exposed thoracic wall was performed using local flap covers from the pectoralis major and rectus abdominis muscle groups. Histopathological examination (HPE) results indicated the presence of a GCT, which showed infiltrative characteristics and the potential for malignancy, although the margins were free from tumor cells.

Postoperatively, the patient was stable and was discharged subsequently on postoperative day seven. The patient was called for follow-up after three months and the scar was found to be healthy with no evidence of recurrence. The patient was asked to come after two weeks for imaging but the patient was lost to follow-up.

## Discussion

**Figure 5:**
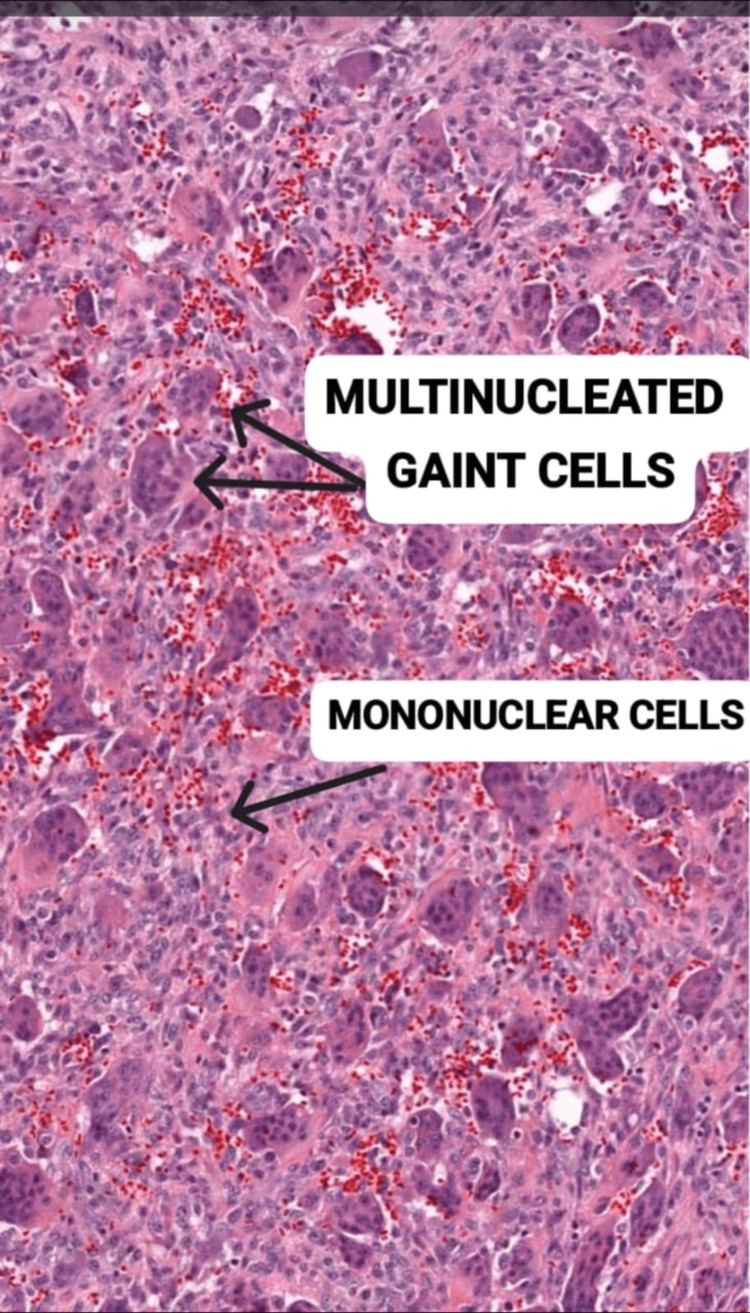
Histopathological image of microscopic giant cell tumor

GCTs are primarily observed in individuals with fully developed bones and are most commonly found in the epiphysis. While the posterior arch is a frequent location for GCTs, they are rare in the rib, with a reported prevalence of less than 1% [5]. Instances involving the anterior arch are even rarer, with very few documented cases in the literature. Generally, GCTs of the bone are uncommon neoplasms, constituting approximately 4-5% of all primary bone tumors [7]. These tumors predominantly affect women, and the tumor cells often express estrogen and progesterone receptors. Histologically, GCTs are characterized by a background of mononuclear stromal cells and large multinucleated cells resembling osteoclasts (Figure 5) [2]. Despite being benign neoplasms, malignant transformation can occur spontaneously or as a result of the tumor's progression from a benign giant cell lesion.

In this case study, we report an unusual development of a massive GCT affecting the outer aspect of the fourth and fifth ribs, which was successfully managed through extensive tumor resection and reconstruction of the chest wall. Despite its benign nature, GCTs are associated with a high rate of local recurrence and potential metastasis to the lungs [8].

Wide excision and chest wall reconstruction have been proposed as effective treatments for GCT originating in the ribs [9,10]. The gold standard approach for managing GCT involves aggressive surgical resection with wide margins [1]. In our case, we performed a surgical procedure to remove the tumor along with a portion of the affected ribs (the fourth and fifth ribs), as well as the adjacent ribs above and below the lesion (the third and sixth ribs). Subsequently, we utilized a PTFE mesh to reconstruct the chest wall, employing the pectoralis major and rectus abdominis muscles to advance a flap cover, as described previously in the literature [11,12].

Histopathological examination revealed no evidence of tumor cells at the margins, with the cells displaying a uniform pattern of spindled stromal cells and numerous large cells resembling osteoclasts. Although metastases from GCT tumors are relatively rare, they exhibit local aggressiveness [3,13]. Approximately 2% of GCT patients may develop pulmonary metastasis, which can occur as solitary or multiple lesions [14]. Thus, there is consensus among clinicians that extensive local excision may reduce the risk of metastasis and improve patient outcomes.

## Conclusions

While GCTs of the anterior arc of the rib are rare, clinicians should maintain a high index of suspicion for malignancy due to the potential for metastasis. Utilizing a combination of clinical evaluation, imaging studies, serum biomarkers, and histopathological examination through biopsy can aid in the accurate diagnosis of GCTs and guide effective treatment and management decisions.
